# Adult NREM Parasomnias: An Update

**DOI:** 10.3390/clockssleep1010009

**Published:** 2018-11-23

**Authors:** Maria Hrozanova, Ian Morrison, Renata L Riha

**Affiliations:** 1 Department of Neuromedicine and Movement Science, Norwegian University of Science and Technology, N-7491 Trondheim, Norway; 2 Department of Neurology, Ninewells Hospital and Medical School, Dundee DD1 9SY, UK; 3 Department of Sleep Medicine, Royal Infirmary of Edinburgh, Edinburgh EH16 4SA, UK

**Keywords:** NREM parasomnias, slow-wave sleep disorders, parasomnias, adult, arousal disorders, review

## Abstract

Our understanding of non-rapid eye movement (NREM) parasomnias has improved considerably over the last two decades, with research that characterises and explores the causes of these disorders. However, our understanding is far from complete. The aim of this paper is to provide an updated review focusing on adult NREM parasomnias and highlighting new areas in NREM parasomnia research from the recent literature. We outline the prevalence, clinical characteristics, role of onset, pathophysiology, role of predisposing, priming and precipitating factors, diagnostic criteria, treatment options and medico-legal implications of adult NREM parasomnias.

## 1. Introduction

Non-rapid eye movement (NREM) parasomnias constitute a category of sleep disorders characterised by abnormal behaviours and physiological events primarily arising from N3sleep [1,2,3] and occuring outside of conscious awareness. Due to their specific association with slow wave sleep (SWS), NREM parasomnias are also termed ‘SWS disorders’.

Behaviours such as confusional arousals, sleepwalking, sleep eating (also called sleep-related eating disorder, or SRED), night terrors, sexualised behaviour in sleep (also called sexsomnia) and sleep-related violence are NREM parasomnias that arise from N3 sleep. Confusional arousals, sleepwalking and night terrors are also termed ‘disorders of arousal’, as they share features of autonomic and motor activation [4].

Research focusing on NREM parasomnias has started to gain momentum only in the last two decades. In recent years, clinicians have started to take an interest in this area, allowing for growth in knowledge on the characteristics, mechanisms, causes and treatment of NREM parasomnias. However, our understanding of this sleep disorder is still far from complete. The aim of this paper is to provide an updated, comprehensive review of adult NREM parasomnias, drawing on recent empirical evidence.

## 2. Prevalence of NREM Parasomnias

The prevalence of NREM parasomnias in the general population varies with age [5] and both children and adults can suffer from these behaviours. Disorders of arousal occur more commonly in childhood than in adulthood, and the prevalence of behaviours typically decreases with age [6]. To illustrate, the prevalence of sleepwalking in children has been estimated at 14.5% [7], whilst the prevalence in adults is around 1.7% [8]. Evidence of childhood experiences of SRED and violence in sleep are lacking—virtually all case studies and reports of these behaviours come from adult patients. Table 1 summarises the prevalence of the different NREM parasomnias, in childhood (overall prevalence) and adulthood (current and lifetime prevalence).

The prevalence studies cited above have utilised different data collection methods and varying sample sizes, giving rise to some methodological limitations (Table 2). For instance, two population studies were conducted by telephone, both by non-clinical staff [8,13]. In the study by Bjorvatn, Gronli and Pallesen [8], only one question was asked about each of the specific parasomnias (e.g., “Have you ever experienced or been told that you have woken up at night in a confusional state without remembering the event the next day?”). Such relatively non-specific questions may be misinterpreted by the respondents, leading to bias in the data. However, with the current lack of validated questionnaires for NREM parasomnias, cross-sectional, simple questionnaires as screening tools can offer some insight into the prevalence of these parasomnias.

Prevalence studies are further limited by the fact that reports of NREM parasomnias are reliant on observation. Therefore, the reported numbers may be underestimated. Population samples often either fail to include bed partners of respondents for corroborating accounts of behaviours, or include those who are single or with no regular bed partner. In these individuals, NREM parasomnias often go unnoticed since amnesia for events is common. In these cases, witness accounts to behaviours are of key importance. The majority of prevalence studies included in Table 1 are subjective and based on retrospective self-report, without clinical assessment. Therefore, these results have to be interpreted with caution.

## 3. Sex Differences in NREM Parasomnias

One study that investigated the full range of NREM parasomnia behaviours in children did not identify any sex differences across behaviours [14]. No significant sex differences were found for confusional arousals, sleepwalking and night terrors in participants over the age of 15 years [15]. Howell, et al. [16] found that 60–83% of all sleep eating reported cases was adult females.

Males experienced sleep talking (no longer included under NREM parasomnias in the ICSD-3) more often than females in one study [17]. Adult males were further found to engage in dangerous, injurious night-time behaviours, violence in sleep, and sexsomnia more frequently than females [18,19,20,21]. However, it is unclear whether sex is associated with nocturnal violence and sexsomnia, or whether the difference lies in referral rates [18].

## 4. Clinical Characteristics of NREM Parasomnias

The different NREM parasomnias share a number of basic features. These include full or partial post-episodic amnesia for events, occurrence of behaviours during the first third of the sleep period, unresponsiveness to external stimuli during episodes, and the influence of various priming and precipitating factors for episodes. However, each of the behaviours also has unique characteristics. Table 3 provides an overview of clinical features and implications of the different NREM parasomnias.

An individual can suffer from more than one of the NREM parasomnia behaviours in a single night [22], which suggests that NREM parasomnias may be different phenotypes of a shared pathophysiology [23]. Simple behaviours are characterised by quiet ambulation, while complex behaviours appear to require the involvement of some executive functions, and are usually of a violent or sexual nature [24].

Notably, most studies that investigate clinical characteristics of NREM parasomnias are based on self-report by patients that visit sleep clinics. The most common complaints among these patients include sleepwalking, night terrors [25], and sleep-related violence [13,19]. Most commonly, patients attending sleep clinics present due to clinical progression of their NREM parasomnia, behaviour of long duration or severe symptoms which may be affecting bed partners/family or the individual themselves. Adult patients presenting with NREM parasomnias at sleep clinics may thus not be representative of other adults with NREM parasomnias in the general population [19].

## 5. Age of Onset of NREM parasomnias

In childhood, NREM parasomnias are common. Paediatric NREM parasomnias involve simple acts, which may occasionally turn into agitated, yet not dangerous or complex behaviours [6]. The origin of paediatric NREM parasomnias has been linked to developmental immaturity of inhibitory neural projections, synapses and receptors. In most cases, the behaviours gradually and spontaneously resolve without intervention during adolescence, as the central nervous system matures [26,27,28]. Thus, paediatric NREM parasomnias are considered self-limiting and are widely perceived as benign by both practitioners and parents. However, NREM parasomnias can also persist into adulthood and in some cases can even start *de novo* in adulthood. Recently, authors of a case study hypothesized hyperthyroidism to be the cause of adult-onset sleepwalking in a patient with no history of the disorder [29]. Apart from this singular case study, the reasons for the variable onset and continuity of NREM parasomnias are unknown.

Irrespective of age of onset, there are some shared clinical characteristics which include: variable amnesia for events, occurrence of behaviours during the first third of the sleep period, unresponsiveness during episodes, and various priming and precipitating factors for episodes. However, behaviours in adulthood can present additional complexities, including scope of the disorder and clinical implications with increased severity. As will become apparent from evidence reviewed later in the manuscript, adults with NREM parasomnias can sleep walk and have confusional arousals as a consequence of sleep deprivation, and behaviours may also involve complex behaviours such as violence, sexualised behaviour and self-harm. While such complex behaviours may be semi-purposeful or inadvertent, in some cases they may have serious medico-legal implications.

Previous research has shown factors that differentiate between childhood- and adulthood-onset of the disorder. Family history of NREM parasomnias is more common in patients with childhood onset of the disorder [18]. One report found that more than 4 out of 5 adult sleepwalkers also had a childhood history of sleepwalking [30]. However, empirical evidence for the continuity of other NREM parasomnia behaviours from childhood to adulthood is currently lacking. In another study, adult-onset patients were found to suffer from neurological co-morbidities twice as frequently as childhood-onset patients [18]. On the other hand, patients who developed NREM parasomnias in childhood and experienced the behaviours continuously into adulthood were found to suffer from more complex [25], more violent and injurious behaviours than those who developed NREM parasomnias in adulthood, with no prior history of the disorder [18,19]. The timing of onset of the disorder therefore seems to predict important clinical characteristics of adult NREM parasomnia patients.

## 6. Psychopathology in NREM Parasomnias

Adult NREM parasomnias have been associated with psychopathology perhaps as long as the sleep disorder has been recognized [31]. Empirical research has established a role for psychopathology that is unique to this patient population [32,33]. A recent review has reported significantly higher rates of NREM parasomnias in patients with psychiatric diagnoses, when compared to individuals with no psychiatric disorders [33]. For example, the lifetime prevalence of adult sleepwalking has been shown to be higher in psychiatric populations than in the general population—8.5% vs. 2%, respectively [34]. Similarly, it has been established that SRED occurs significantly more often in inpatients with eating disorders than in the general population, prevalence rates being 16.7% vs. 4.6% [10].

Evidence suggests an association between NREM parasomnias and mood and anxiety disorders [35]. Subjective self-report measures have shown that roughly 16–25% of adult sleepwalkers also suffer from a depressive disorder or bipolar disorder [15,18,32]. By comparison, the global prevalence of people estimated to suffer from depression was 4.4% in 2015 [36] and 2.4% for bipolar disorder [37]. These findings hold importance for clinical practitioners, and may have implications for the management and treatment of both the sleep disorder and the psychological co-morbidity.

While the core symptomatology of NREM parasomnia patients with or without psychopathology remains the same, a recent study has identified unique clinical features in adult NREM parasomnia patients with high levels of psychopathology, as measured by self-report questionnaires. These included a decreased likelihood of a family history of NREM parasomnias and a higher frequency of nightmares [32]. In a psychiatric population, adult-onset NREM parasomnia patients experienced a higher frequency of episodes, with more varied and complex clinical characteristics, when compared to childhood-onset patients. The adult-onset patients in this sample had a higher incidence of sleep-related eating disorder and insomnia was closely associated with adult sleepwalking [34]. Co-morbidity between adult NREM parasomnias and psychopathology may change a patient’s clinical profile.

Triggers specific for adult NREM parasomnia patients with psychopathology have been identified. These included pain, migraines, and in some cases, caffeine—factors known to fragment sleep [32]. These findings give valuable insight into the functional aspects of NREM parasomnias in patients with psychopathology. Clinicians should be aware that these specific triggers might increase the likelihood of experiencing an episode of NREM parasomnia. When given appropriate guidelines and medical advice, patients may learn how to avoid these triggers and thus reduce the likelihood of them experiencing a nocturnal episode.

It is important to note that studies exploring psychopathology in adult patients with NREM parasomnias have yielded contrasting results. For instance, research has shown that in patients with sleepwalking and night terrors, who presented with a psychiatrically diagnosed mental health condition (depression or major anxiety), treatment of the psychiatric condition by counselling, psychotherapy and/or pharmacotherapy did not alleviate the NREM parasomnia symptoms [38,39]. However, it is also important to recognise dissociation as a potential differential diagnosis for NREM parasomnias and its association with psychiatric disorders. Differentiating between the two is challenging and may complicate existing work in this area. The exact role of psychopathology in NREM parasomnias is therefore still to be established, and future research should systematically evaluate the clinical significance of this association, as current evidence is limited.

## 7. Pathophysiology of NREM Parasomnias

Episodes of NREM parasomnias are characterised by abnormal nocturnal motor behaviours with the absence of conscious awareness. The person is asleep, yet their eyes are open, they are moving around and experiencing autonomic arousal. The mechanism of this mixed state of being is, despite more than 60 years of investigation, still not fully known.

The predominant neurobiological hypothesis states that NREM parasomnias result from a state dissociation between wake and NREM sleep. This is thought to form the basis of arousal disorders. Three main types of data acquisition have been utilised to elucidate the neurobiology of NREM parasomnias: electroencephalography (EEG), functional imaging, and structural imaging studies.

### 7.1. Electro-Encaphalographic (EEG) Studies

The first line of evidence came from an intracerebral stereotactic EEG study by Terzaghi, et al. [40]. During an episode of NREM parasomnia in a patient, wake-like beta activity to motor and cingulate cortices was recorded. Furthermore, there was an increase in delta activity, typical of N3 sleep, in frontoparietal associative cortices. Supporting lines of evidence came from another EEG study, which utilised low resolution electromagnetic tomography to show increased current density in beta bands in the cingulate motor area immediately prior to an episode of sleepwalking [41]. The enhancement of N3 sleep in the frontoparietal areas, versus the wake-like activation of motor and cingulate cortices suggests dissociation between the states of wake and sleep.

Other studies provided three pieces of evidence for the co-existence of N3 and arousal prior to an episode of sleepwalking. Firstly, posterior areas saw a decreased connectivity in the delta frequency band. Secondly, the anteroposterior network saw increased functional connectivity in alpha and beta frequency bands. Lastly, increased spectral power in delta and theta frequencies was observed [42]. Together, these studies suggest that sleep and wakefulness may co-exist in the brains of adult NREM parasomnias patients.

Furthermore, localised sleep differences measured by scalp EEG were also observed during sleep without any behavioural episodes in NREM parasomnia patients. A decrease in slow wave activity power was observed in the cingulate, motor and sensorimotor associative cortices of patients, as compared to healthy controls. Importantly, these changes were not only present during NREM sleep, but also during REM sleep and wakefulness. These results give rise to the possibility of trait-like functional changes in neuronal excitability in patients with NREM parasomnias [43].

### 7.2. Functional Imaging Studies

Imaging studies have identified specific patterns of activity during SWS in healthy subjects. An overall reduction in brain metabolism when compared to wakefulness is the most striking characteristic [44]. This reduction is most obvious in brain regions responsible for a range motor and cognitive functions: brainstem, thalamus, basal ganglia, basal forebrain, prefrontal cortex, anterior cingulate cortex and precuneus [44,45]. As functional imaging studies have shown, there are some crucial differences in the characteristics of SWS in healthy controls and NREM parasomnia patients. All in all, these findings complement the functional EEG findings of Terzaghi and colleagues [40] and Desjardins and colleagues [42], which point to the concomitant presence of wake and sleep associated with NREM parasomnias.

Functional imaging studies have also shown that during an arousal parasomnia, local brain phenomena exhibit characteristics typical for both wakefulness and NREM sleep, simultaneously [45,46]. Single photon emission computed tomography (SPECT) was used to investigate brain activation during an episode of sleepwalking [46]. This study revealed two findings that point to state dissociation: activation of the posterior cingulate cortex and anterior cerebellum, and a decrease in regional cerebral blood flow in the frontoparietal associative cortices. Concurrently, the usual deactivation of the thalamus [47] was not present. During normal SWS sleep, the thalamus and cingulate cortex are deactivated, which points to abnormal processes in these regions during sleepwalking. Symptoms such as post-episodic amnesia and absence for conscious awareness are likely associated with the lack of regional cerebral blood flow to the prefrontal cortices.

Another SPECT study investigated whole brain perfusion patterns in sleepwalkers and controls after a night of total sleep deprivation, during sleep and resting-state wakefulness [48]. The analysis of SWS showed that when compared to controls, sleepwalkers exhibit reduced regional cerebral perfusion in the frontal (middle frontal and medial frontal gyri) and parietal areas (left postcentral gyrus). These regions have previously been associated with the generation of SWS and NREM parasomnia episode occurrences. Regional decreases in brain perfusion were also found in the insula and superior temporal gyrus. The reduction of brain perfusion in these areas may be responsible for the impaired awareness and poor judgement typical of NREM parasomnia episodes. Interestingly, reduced brain perfusion in frontal and parietal regions persisted during resting-state wakefulness. These findings replicate the evidence from an earlier study [45], which hypothesised these areas to be responsible for the daytime impairment experienced in NREM parasomnia patients.

### 7.3. Structural Imaging Studies

One recent structural imaging study aimed to identify whether the grey and white matter morphometry of NREM parasomnia patients differs in any way from normal subjects [49]. The study provided supplementary evidence of structural changes in the regions implicated in the functional imaging studies, suggesting that the functional changes found in NREM parasomnias might be underpinned by specific neuro-anatomical substrates. In the study, diffusion tensor imaging in adult patients with NREM parasomnias was utilised, and results were compared to healthy controls. Results identified the grey matter of the left dorsal posterior cingulate cortex and posterior mid-cingulate cortex to be of significantly smaller volume in patients. The posterior mid-cingulate grey matter reductions might portray the neuro-anatomical substrate for the differential activation states of the motor and cingulate cortices that were identified by previous studies as giving rise to the engagement in motor behaviour during episodes of NREM parasomnias [40]. In addition, the inadequate deactivation of the frontoparietal association cortices might elucidate the varying states of awareness from the environment that often accompany behaviours such as sleepwalking [23].

The above studies demonstrate that sleep and wakefulness can coexist in the brain. This occurs by means of an apparent conflict in activation: by arousal of the emotion, motor and executive function-related cingulate cortex and the movement-related cerebellum and motor cortex, with simultaneous deactivation of the associative cortices. However, these results do not explain why certain NREM behaviours occur in some people but not in others, and what mechanisms enable the co-occurrence of two or more NREM parasomnias in one person. Further research needs to replicate these results, and to elucidate whether this state dissociation is unique solely to NREM parasomnias. Empirical studies are needed to explore which specific triggers and causative factors are involved.

## 8. Predisposing, Priming and Precipitating Factors of NREM Parasomnias

NREM parasomnias occur only under specific conditions, whenever factors that predispose, prime and precipitate the behaviours interact, and together create the ‘ideal’ conditions for the behaviour to occur. Predisposing factors include genetics and family history; priming factors include conditions that increase the proportion of deep NREM sleep per night or that increase the arousal threshold; and precipitating factors include specific conditions that trigger the onset of the episodes. In the absence of these factors, an episode of NREM parasomnia is unlikely to occur.

### 8.1. Predisposing Factors

The occurrence of NREM parasomnias in families, observed in clinical practice and in a small number of original studies [19,50], point to a possible common genetic element for NREM parasomnias. In one study, the likelihood of a child manifesting NREM parasomnia behaviours was 22% if neither of the parents had the disorder; 45% if one parent had the disorder; and 60% if both parents were affected by the sleep disorder [51]. Furthermore, the concordance for adult sleepwalking was found to be five times higher in monozygotic than in dizygotic twins [30]. These results point to the crucial role of genetics in NREM parasomnias. However, it is important to note that genetics alone cannot cause the sleep disorder to occur but rather, increases susceptibility to experiencing the disorder in the ‘correct’ setting. One study postulated that the propensity to experience a NREM parasomnia was inherited in an autosomal dominant fashion [52].

More recent work has also examined the role of the HLA system, specifically HLA DQB1*05:01 [53,54], and its association with NREM parasomnias. The initial study showed that 35% of sleepwalkers carried the genotype, compared to 13.3% of healthy controls in a Caucasian sample [54]. This finding was extended by Heidbreder and colleagues [53], who investigated the incidence of the HLA DQB1*05:01 genotype not only in sleepwalkers, but also in those with sleep terrors and confusional arousals. The allele was found in 41% of NREM parasomnia subjects. However, this does not account for the remaining 59% of patients in this cohort. Furthermore, 42% of the patients in this study had a positive family history for NREM parasomnias.

### 8.2. Priming Factors

In those who have a susceptibility to experiencing NREM parasomnias, factors that increase the proportion or depth of N3 NREM sleep per night, factors that increase the arousal threshold and / or factors that make NREM sleep more unstable, are thought to elevate the odds of experiencing an episode [55]. The next sections will outline how the abnormalities in NREM structure, sleep deprivation, stress and trauma, as well as substance use prime the occurrence of episodes in NREM parasomnia patients.

#### 8.2.1. Abnormalities in NREM Structure

Because the proportion of N3 sleep is thought to be at its peak in childhood and then decreases from adolescence onwards [51,56], it has been hypothesized that for adults to experience NREM parasomnias, the proportion of N3 sleep has to be greater than the adulthood average. Previous research has investigated the proportion of N3 sleep in NREM parasomnia patients when compared to healthy subjects, and found the proportion of N3 sleep to be significantly greater in patients than in healthy controls [2]. However, other studies showed no statistical difference [57]. The reason for such conflicting and inconclusive results may be that no specific cut-off value to distinguish between a normal and elevated amount of N3 sleep has been established, underlining the methodological issues present. In a number of studies, analysis of polysomnography (PSG) recordings showed an increased frequency of arousals from N3 sleep in those suffering from NREM parasomnias when compared to healthy controls [2]. The reason for this increased rate of arousals is unknown; some have suggested they are caused by pathological NREM instability [58], while others have hypothesized that it could be a genetic trait associated with NREM parasomnias [59]. However, other research investigating the rate of arousals from N3 sleep produced conflicting findings which showed no differences in sleep macrostructure between those with NREM parasomnias and normal controls [57]. A large group of subjects with no diagnosis of NREM parasomnias also had a high number of arousals out of N3 sleep. These arousals were associated with apnoeas and hypopnoeas [60]. Therefore, the exact role played by arousals out of N3 remains open for investigation. It is important to note that at present, there is no data across the general population that specifies the normal number of arousals out of N3 per night.

#### 8.2.2. Sleep Deprivation

Sleep deprivation intensifies the pressure for N3 sleep [61]. In healthy individuals, enhancing the pressure for N3 sleep makes for a more consolidated NREM sleep period [62]. Together with the proposition that NREM parasomnias are more likely to occur when the proportion of N3 sleep increases, sleep deprivation became a strong point of interest when investigating it as a potential primer of NREM parasomnia episodes. As a result, several studies explored the hypothesis that sleep deprivation might be a primer for NREM parasomnias. The evidence is currently conflicting. On one hand, studies found that, contrary to healthy sleepers, the recovery sleep of patients with NREM parasomnias is more fragmented. Indeed, the fragmentation results in nocturnal episodes of NREM parasomnias [63]. These results show that sleep deprivation elevates the likelihood of the behaviours occurring in predisposed individuals. On the other hand, one study did not replicate these results, and instead showed no effect of sleep deprivation on the number of NREM parasomnias during recovery a night [64].

A number of methodological inconsistencies may have contributed to the above-mentioned results. The sleep deprivation period in these studies ranged between 24 to 38 h. PSG recordings were done during the daytime in some participants, during the night in others, and some had baseline recordings during the day and recovery recordings during the night. Circadian rhythm effects were thus largely ignored. Due to the considerable variation in the methods used, the reliability of these between-study comparisons is questionable. To obtain better, more reliable results, future research should establish a standardised protocol for testing the effects of sleep deprivation in patients with NREM parasomnias.

#### 8.2.3. Stress and Trauma

The proposition that in some individuals, sleep disorders might be stress and trauma related has received some attention [65,66]. Based on clinical practice, there certainly seems to be an association between highly stressful and traumatic events and either the initiation or exacerbation of NREM parasomnia episodes. A number of studies have shown that patients report psychological stress or stressful events as triggers that both prime (chronic stress) and precipitate (acute stress) episodes [19,25,32,55]. In a recent PSG study of adult NREM parasomnias, stress was identified as a trigger by two-thirds of those that reported being aware of having triggers for their episodes [18]. However, there is an urgent lack of systematic studies identifying the causational relationship between subjective and objective stress, and the incidence and intensity of NREM parasomnia episodes.

Agargun and colleagues [14] found that the majority of young adults who reported sleep-related violence had endured traumatic experiences in their past. It should be noted that the subjects in this study were not formally diagnosed with NREM parasomnias. Mysliwiec, et al. [67] reported a prominent rise in night terrors and sleepwalking in the Department of Defence and Medical Treatment facilities in the US Army—a population with high exposure to stress and traumatic events. This evidence suggests that trauma might play a role in these abnormal nocturnal behaviours. The nature of this association is understudied, and to date, no direct relationship has been established between NREM parasomnias and traumatic experiences.

#### 8.2.4. Medication

A large number of case reports documenting NREM parasomnia episodes following the intake of various types of medications exist (reviewed in [59,68]). The medications cover nearly all psychotropics, but fall primarily into five main classes: non-benzodiazepine sedatives such as Zoldipem [69], benzodiazepines such as Temazepam or Diazepam [70], antidepressants such as Citalopram or Amitriptyline [71], beta-blockers such as Propanolol [72], and mood stabilisers such as Lithium [73]. However, methodological limitations exist in studies investigating the effects of medication on the incidence of NREM parasomnia episodes. The majority of studies investigating this association comprise of cases of patients with complex medical and / or psychiatric conditions, who often take a number of additional medications of varying doses. Indeed, it has been suggested that non-benzodiazepine sedatives may precipitate sleepwalking in psychiatric patients more often than in other patients [74]. It is possible that a bias in research and clinical practice exists, by which closer monitoring of psychiatric patients than other patients results in elevated rates of reported medication-related episodes in this population. Another reason may also be that interactions between non-benzodiazepine sedatives with other drugs that increase the risk of sleepwalking exist, and together, this interaction leads to a higher incidence of NREM parasomnia episodes [68].

There is a dearth of controlled, randomised trials investigating the effects of medications in patients clinically diagnosed with NREM parasomnias. Only the non-benzodiazepine sedatives, specifically zolpidem, have demonstrated causation between the drug and sleepwalking in a clinical trial [75]. For classical benzodiazepines, antidepressants, beta-blockers and mood stabilisers, evidence is based on case studies. Although case studies are useful in allowing rare phenomena to be studied, and provide hypotheses for the specific contexts, they do not allow for the identification of specific causation between the different drugs and the nocturnal behaviours. Future research should therefore focus on designing controlled, randomised clinical trials to provide direct evidence of medication-induced NREM parasomnia episodes.

#### 8.2.5. Alcohol

The disruptive effect of alcohol on sleep is well established and accepted. However, there has been considerable debate about its association with NREM parasomnias [76]. Whenever considering its role in abnormal nocturnal behaviours, it is important to first establish two important factors: the amount and the regularity of alcohol intake. Even a single dose of alcohol influences sleep architecture: when compared with controls, subjects had rapid sleep onset, well-consolidated sleep in the first half of the night with more N3 sleep. The onset of REM was delayed. In addition, sleep in the latter half of the night was more fragmented. At high doses of alcohol, sleep onset latency was reduced and an increase in N3 sleep could be consistently seen across the whole night. Again, REM sleep was reduced in the first part of the night [77]. These results clearly indicate that alcohol does have an influence on sleep architecture and on the proportion of NREM sleep.

The effects of alcohol on sleep differ dramatically between occasional and regular drinkers. Following intoxication in occasional drinkers, sleep onset latency is reduced, and in the first half of the night, the proportion of N3 sleep increases and REM sleep decreases. In the second half of the night, sleep is fragmented and light, the proportion of REM sleep increases and awakenings are more frequent [78]. In both abstaining [79] and active alcoholics [80], N3 sleep is decreased and the proportions of N1 and REM sleep increase.

It is possible that alcohol could prime abnormal behaviours arising from N3 sleep in predisposed individuals, if precipitating conditions are satisfied. Indeed, 92% of NREM parasomnia patients who consumed alcohol regularly reported that alcohol increased the incidence of their episodes [81] and influenced their sleep quality and symptoms [19,82]. One recent study surveyed the opinions of sleep medicine experts on this issue, and revealed that a clear majority of sleep experts agreed that alcohol could trigger an episode of NREM parasomnias [83]. These studies show support for the hypothesis that alcohol may act as a trigger for NREM parasomnias, but further evidence is needed to elucidate the causality and mechanisms underpinning the effects of alcohol on NREM parasomnias.

### 8.3. Precipitating Factors

The occurrence of predisposing and priming factors alone is not enough to cause an episode of NREM parasomnia. Typically, an additional factor needs to be present to set the behaviours off—a precipitating trigger that interacts with the predisposing and priming factors. Research assessing the role of precipitating factors is scarce, as there seems to be considerable within- and between-persons variability in how these manifest [84]. Among the known precipitating factors are co-morbid sleep disorders such as sleep disordered breathing and periodic limb movements, and environmental factors such as noise and touch.

#### 8.3.1. Other Sleep Disorders

Microarousals secondary to apneas, hypopneas and related breathing events in those with sleep disordered breathing or obstructive sleep apnea/hypopnea syndrome (OSAHS) were shown to precipitate episodes of NREM parasomnias, in both children [85] and adults [38]. Similarly, arousals associated with excessive movements in those suffering from periodic limb movements (PLM) were shown to elicit NREM parasomnias [85]. This is problematic especially when the co-morbid sleep disorder is not properly diagnosed and is left untreated. In such cases, treatment of the NREM parasomnia itself is rarely effective. On the other hand, it has been shown that treatment of the co-morbid sleep disorder resolves the abnormal behaviours associated with NREM parasomnias [85].

#### 8.3.2. Environmental Factors

Clinical practice along with a number of laboratory studies has reported environmental factors such as external noise or touch as a direct trigger of NREM parasomnias [86,87]. In a controlled sleep laboratory setting, noises have been shown to trigger episodes of NREM parasomnias in predisposed individuals primed by sleep deprivation [87]. In this study, a combination of predisposing and priming factors gave rise to NREM parasomnia episodes in patients but not in controls, a finding of important clinical diagnostic utility. Another triggering factor identified in clinical practice is a change in sleeping environment from a familiar one to one that is novel and foreign. This precipitating factor may occur less frequently, but seems to play an important role in increasing the frequency of episodes in NREM parasomnia patients who sleep in unfamiliar places, when visiting friends or travelling for business or pleasure [88]. Further research needs to be undertaken to establish the role of other environmental factors or arousing external stimuli that act as triggers of NREM parasomnias in the presence of predisposing and possibly other priming factors.

## 9. Diagnosing NREM Parasomnias

Unlike other sleep disorders (e.g., REM behaviour disorder) for which objective diagnostic PSG protocols exist, NREM parasomnias are generally diagnosed solely on the basis of clinical interview which is subjective on the part of the patient. Collateral history obtained from a patient’s bed partner or parent can be invaluable. There are a number of diagnostic criteria for NREM parasomnias in existence established in the context of three international classification systems: ICSD-3 [5], the Diagnostic and Statistical Manual of Mental Disorders 5 (DSM-5) [1] and the International Classification of Disorders 10 (ICD-10) [89]. The specific criteria outlined in these classification systems are outlined in Table 4.

Unfortunately, there are many inconsistencies across the different diagnostic criteria, which pose challenges in clinical practice. At present, the ICSD-3 is the most widely used diagnostic manual in sleep medicine. It offers more depth and specificity for the assessment of the different NREM parasomnia behaviours, unlike the ICD-10, which is limited in the recognition of the more complex behaviours such as sleep eating, sexualised behaviours in sleep, and violence in sleep. For instance, the ICSD-3 mentions the possibility of nocturnal violence for disorders of arousal, but does not acknowledge sleep-related violence to be a separate pathophysiological subtype. Similarly, violence is only mentioned for sleepwalking in DSM-5, and is not recognised at all in the ICSD-3.

Despite it not being necessary for the diagnosis of NREM parasomnias, polysomnography (PSG) is often used to screen for other potential concomitant sleep disorders, which might be worsening the nocturnal episodes. During a PSG assessment, close attention is paid to any evidence of sleep disorders such as REM behaviour disorder, overlap parasomnias, sleep apnoea (OSA) or periodic limb movements in sleep (PLMS). Furthermore, PSG is used to differentiate NREM parasomnias from nocturnal frontal lobe epilepsy (otherwise known as sleep-related hypermotor epilepsy [90]) or complex partial seizures, which may have similar symptoms and atypical motor behaviours [5].

During a laboratory PSG assessment used for differential diagnosis, an episode of NREM parasomnia may occur. Although this occurrence is rare [91], the ICSD-3 [5] states that when PSG does identify arousals out of N3 sleep (usually in the form of confusional arousal, very rarely out-of-bed attempts), this can be considered a contributing factor to the clinical diagnosis of NREM parasomnias. A number of recent studies reported, somewhat surprisingly, episodes of NREM parasomnias in roughly 60% of the monitored patients during a night of PSG monitoring [92,93]. It remains unclear why some studies find a low prevalence of episodes during laboratory PSG assessments, while others find a high prevalence.

Although there is a lack of formal diagnostic protocols, a scale for assessing the severity of arousal disorders, the Paris Arousal Disorders Severity Scale (PADSS), has recently been developed by Arnulf et al. [94]. The scale has powerful psychometric properties, as well as valid and reliable subscales. It is self-administered and therefore useful in patient surveys and interventional research. It may be used by general practitioners to aid with the diagnostic process, the identification of complex behaviours and ultimately with referral to sleep medicine specialists. It also provides a means to assess the efficacy of new drug and intervention treatments, as well as changes over longer periods of time, making it an useful tool in both clinical practice and research. However, it does not cover any of the possible psychological issues faced by patients with NREM parasomnias and may not distinguish between dissociative episodes, nocturnal epilepsy or SWS disorders consistently.

Taken together, there is a lack of objective, validated, standardised and quantitative diagnostic criteria for NREM parasomnias. Until diagnostic tools for these qualities are developed and utilised in the area of sleep medicine, the accuracy of the diagnostic process and the extent to which research will be able to close the current gaps in our understanding of NREM parasomnias, remains uncertain.

## 10. Treatment of NREM Parasomnias

For adults and children with simple NREM parasomnias, treatment is often unnecessary as the behaviours are benign and do not pose a risk to the patient or family members/cohabitees. For adults with more complex behaviours, the first-line of intervention is to offer advice on safety, sleep hygiene and stress management. To decrease the risk of NREM parasomnias, the affected patient should ensure his/her sleeping place is safe and secure. In the case of sleepwalking, all sharp objects and furniture not mounted to the walls should be removed, as well as any objects the individual could trip over. Windows should be locked, and alarms should be put on doors if there is a risk of using these and wandering outdoors. Furthermore, it is important that the affected patient and their bedpartner or family understand the role of priming and precipitating factors, so that sleep deprivation, environmental disturbances and stress can be reduced to a minimum. In a recently studied cohort of 512 patients with NREM parasomnias, an intervention utilising advice on safeguarding and sleep hygiene successfully alleviated and resolved the symptoms in 12.9% of the sample [95]. For patients with more severe clinical symptoms, pharmacological and / or psychological therapy may be additionally needed to control the symptoms. These forms of therapy are used for patients with increasing intensity and frequency of episodes, for patients with complex and dangerous behaviours, and for patients who experience significant daytime functional impairment caused by their nocturnal behaviours. Reports show that pharmacological therapy is used much more often than psychological therapy (78.4% vs. 7.8%), and that both types of therapy have high success rates in treating the symptoms [84].

### 10.1. Pharmacological Therapy

In clinical practice, pharmacotherapy has long been the preferred treatment for patients with complex clinical histories, recurrent and disruptive behaviours [96]. So far, the benefits of pharmacological treatment have been evidenced mostly by clinical experience, uncontrolled case studies, and a handful of small trials (for a review, see [97]). A recent study analysed the pharmacological treatment approach in 512 patients with NREM parasomnias, and yielded valuable results. The benzodiazepine drug, clonazepam, was most commonly used and successfully alleviated symptoms in 72.2% of patients. Since benzodiazepines were not suitable for all candidates in this study, other drugs were prescribed. These included the non-benzodiazepine sedative zolpidem, various antidepressants, such as fluoxetine, citalopram and mirtazapine, as well as melatonin. The success rate of these varied from 58–77% [84].

### 10.2. Psychological Therapy

Psychological therapies are used in patients who do not wish to take medication, experience unpleasant side effects, those who wish to engage in therapy in combination with medication, or in those who do not find medication useful. Psychological therapies are especially useful in those whose episodes are triggered by stress or other emotional and psychological problems, which may be determined through psychological evaluation with the use of personality and symptom scales, as well as psychotherapy [98]. Clinical experience and case reports show that psychotherapy [98], behavioural and cognitive-behavioural therapy [99], mindfulness-based stress reduction [84], and hypnotherapy [100] improve the symptoms and manifestations of NREM parasomnias in some patients, but not others.

The pronounced lack of systematic, adequately powered, appropriately controlled and randomised trials on the efficiency of the various pharmacological and psychological treatments offered are striking. Awareness has to be raised about the lack of evidence, and both the clinician and the patient should keep this in mind when deciding on the most appropriate line of treatment.

### 10.3. Therapy of a Concomitant Sleep Disorder

The co-occurrence of another sleep disorder, such as OSA, may be present in NREM parasomnia patients. Research has shown that treatment of the co-morbid disorder, even of its mild forms, resolved the abnormal nocturnal behaviours associated with NREM parasomnias [38,84]. In these patients, continuous positive airway pressure for OSA should be considered as the first line of treatment, before resorting to pharmacological or psychological therapy for the treatment of the NREM parasomnia. These results underline the importance of utilising PSG to diagnose concomitant sleep disorders in NREM parasomnia patients, as recommended by the ICSD-3.

## 11. Medicolegal Issues in NREM Parasomnias

The importance of reliable empirical studies investigating the pathophysiology of NREM parasomnias, as well as clear diagnostic guidelines, are highlighted when addressing the complex instances of nocturnal NREM parasomnia behaviours with medicolegal implications. Legal practice has seen cases in which the diagnosis of NREM parasomnias was used as a defence in crimes such as murder, rape, road traffic accidents and attempts to conceal forensic evidence [101]. However, it is often difficult to distinguish between a disorder of sleep, dissociative behaviours or deliberate behaviour with denial of recall. With all the limitations discussed in this review, especially in terms of establishing the correct diagnosis and appropriate investigations, it is clear this is a challenging area to present in courtroom setting.

## 12. Conclusions

In this updated overview of NREM parasomnias, we have focused on what is known about this disorder but also highlighted areas where our knowledge is still deficient, not least because the definitions of the disorder remain disparate across specialties.

The differential diagnosis for NREM parasomnias remains wide, particularly so for adults compared to children due the variability of presentations, the lack of collateral history in many cases and the poor recognition in many sleep clinics of psychiatric and other sleep disorders that can act as mimics. The rationale for investigating and treating NREM parasomnias presenting to a clinic include safety of the individual and co-sleepers, amelioration of personal distress regarding the disorder and the safeguarding of the individual within a medico-legal framework.

We are no closer to elucidating the aetiology of the disorder, although our understanding of the physiological sleep-wake states occurring within it is improving. Currently, we have limited resources at our disposal in terms of accurately phenotyping the disorder. We believe that a unitary definition with strict criteria should be developed based on objective as well as subjective data. There are no large, randomised, controlled trials in the management of NREM parasomnias and these would need to be predicated on excellent phenotyping, also taking precipitating factors into account in order to best tailor therapy for the individual sufferer. However, we are slowly but surely moving towards these goals and co-operation across research groups internationally should be fostered and supported.

## Figures and Tables

**Table 1:** Current and lifetime prevalence of the different types of NREM parasomnias.

NREM Parasomnia	Prevalence of Symptoms		
	Childhood	Current Prevalence	Lifetime Prevalence
Confusional arousals	17% [5]	6.9% [8]	18.5% [8]
Sleepwalking	14.5% [7]	1.7% [8]	6.9% [9]–22.4% [8]
SRED	-	2.2% [8]	4.5% [8]
		16.7% [10]	
Night terrors	17.3% [11]–39.8% [7]	2.7% [8]	10.4% [8]
Sexsomnia	-	2.7% [8]–6% [12]	7.1% [8]

**Table 2:** Type of article, number of participants and method of data collection used in NREM parasomnia prevalence studies.

Reference	Type of Article	N	Method of Data Collection
[7]	Longitudinal study	N = 9142	Parent-reported behavior
[8]	Population based cross-sectional study	N = 1000	Telephone interview
[9]	Systematic review and meta-analysis following PRISMA guidelines	51 studies included, N = 100490	Varied
[11]	Longitudinal study	N = 1353	Parent-reported behavior
[10]	Cross-sectional study	N = 700	Self-report questionnaire
[12]	Review of online surveys	Overall N not reported	Varied
[13]	Cross-sectional study	N = 19961	Telephone interview

**Table 3:** Complexity and clinical characteristics of the different types of NREM parasomnias.

NREM Parasomnia	Clinical Implications	Clinical Features
Confusional arousals	Minimal	Individuals may sit up in bed, look around in a confused manner. Behaviour may progress to sleepwalking if subject leaves the bed
Sleepwalking	Minimal to moderate	Walking in the bedroom, simple searching behaviours, or jumping out of bed with a startle
	Moderate to severe	Behaviours that seem to engage executive functioning: climbing on a chair to change lightbulbs, driving, moving furniture
SRED	Moderate	Involuntary consumption and preparation of food and drink during the night, consumption of bizarre foods or inedible items, involves hazardous activities such as handling knives
Night terrors	Severe	Sudden episodes of intense fear and dread, frequently accompanied by screaming and / or lashing out in a protective manner as a result of upsetting dream mentation or imagery, heightened autonomic function. Commonly overlap with sleepwalking
Sexsomnia	Moderate to severe	Sexual arousal with autonomic activation, attempted and / or forced sexual intercourse, groping of bed partner, masturbation or vocalisations, often unusual for the patient in terms of partner, intensity or sexual act

**Table 4:** The diagnostic criteria of the currently used classification systems International Classification of Sleep Disorders 3, Diagnostic and Statistical Manual of Mental Disorders 5 and International Classification of Disorders 10.

Diagnostic Manual	Category	Disorder	Diagnostic Criteria
**ICSD-3**	NREM-related parasomnias > Disorders of Arousal (From NREM Sleep)	Confusional arousals	Recurrent episodes; inappropriate or absent responsiveness during episodes; minimal or no dream recall; partial or complete amnesia for events; not better explained by another sleep, mental or medical condition, medication, substance use
		Sleepwalking	
		Night terrors	
	NREM-related parasomnias > Clinical and pathophysiological subtype of NREM-related parasomnias	Sleep related abnormal sexual behaviours	
	NREM-related parasomnias	Sleep-related eating disorder	Repeated episodes of abnormal eating subsequent to an arousal from the main sleep period. At least one of the following is present: consumption of inappropriate foods; potentially injurious behaviours, adverse health consequences. Partial or complete amnesia for events; not better explained by another sleep, mental or medical disorder, medication, substance use
	Isolated symptoms and normal variants of parasomnias	Sleep talking	Sleep talking, with varying degrees of comprehensibility
**DSM-5**	NREM	Sleepwalking	Recurrent episodes; minimal or no dream recall; amnesia for events; causes marked distress or impairment in essential areas of functioning; not better explained by another mental or medical condition, medication, substance use
		Sleepwalking with sleep-related eating	
	Sleep Arousal Disorders	Sleepwalking with sleep-related sexual behaviour	
		Sleep terrors	
**ICD-10**	Behavioural syndromes associated with physiological disturbances and physical factors, mental and behavioural disorders > Nonorganic sleep disorders	Sleep walking	Episodes typically during the first third of sleep period, relative unresponsiveness to external stimuli, amnesia for events
		Night terrors	
